# Essential Oils from Selected Mediterranean Aromatic Plants—Characterization and Biological Activity as Aphid Biopesticides

**DOI:** 10.3390/life13081621

**Published:** 2023-07-25

**Authors:** José Luis Casas, Aitor Sagarduy-Cabrera, María López Santos-Olmo, Mª Ángeles Marcos-García

**Affiliations:** Research Institute CIBIO (Centro Iberoamericano de la Biodiversidad), Scientific Park, University of Alicante, Ctra. San Vicente del Raspeig s/n, E-03690 San Vicente del Raspeig, Alicante, Spain; aitorsc98@gmail.com (A.S.-C.); maria.lopezs@gcloud.ua.es (M.L.S.-O.); marcos@ua.es (M.Á.M.-G.)

**Keywords:** biopesticide, integrated pest control, natural insecticide, *Rhopalosiphum padi*, sustainable agriculture

## Abstract

The need for alternatives to synthetic pesticides is a priority today, especially when these pesticides are directed against aphids, one of the more challenging pests facing modern agriculture. Essential oils may be one of these alternatives. We assayed the insecticidal potential of essential oils from *Thymus vulgaris*, *Rosmarinus officinalis* var. ‘prostratus’ and *Lavandula dentata*. Essential oil extraction was carried out by hydrodistillation in a Clevenger-type apparatus for 3 h and their respective composition was elucidated by gas chromatography-mass spectrometry. The essential oil fraction from *T*. *vulgaris* contained 81.20% monoterpenoids and 12.85% sesquiterpenoids; *R*. *officinalis* var. ‘prostratus’ contained 91.98% monoterpenoids and 1.93% sesquiterpenoids, while *L. dentata* contained 69.60% monoterpenoids and 8.05% sesquiterpenoids. The major components found were 1,8-cineole (18.11%), camphor (11.18) and borneol (10.32%) in *T*. *vulgaris*; α-pinene (18.72%), verbenone (13.42%) and 1,8-cineole (10.32%) in *R. officinalis*; and 1,8-cineole (34.65%), camphor (7.58%) and β-pinene (6.39%) in *L*. *dentata*. The insecticidal activity of the essential oils was evaluated by contact toxicity bioassays against the bird cherry oat aphid, *Rhopalosiphum padi* L. We observed a mortality rate of 78.3% ± 23.9 at 15 μL/mL with *T. vulgaris*, 54.7% ± 25.8 with *L*. *dentata* (although at a lower concentration, 10 μL/mL), and 56.7% ± 25.6 at 15 μL/mL with *R*. *officinalis*. Our results suggest that thyme essential oil may be particularly promising for integrated aphid management provided that specific conditions of use and dosages are observed.

## 1. Introduction

Aphid control in an agricultural environment is particularly difficult as a result of two main factors. The first is the nature of the pest itself. Aphids are a species-rich group of insects included in Aphidoidea (Hemiptera), a superfamily comprising Adelgidae, Phylloxeridae and Aphididae, which are parthenogenetically viviparous [1]. This mode of reproduction converts these r-strategists into a very serious threat to crops as they are capable of attaining very high population densities in relatively short times [2]. The second reason derives from the peculiar feeding habit of these insects. Aphids feed on phloem sap using specially adapted mouthparts, the stylets, to penetrate plant tissues in search of sieve elements and incorporate nutrients in the form of photoassimilates without inflicting serious damage to host plant [3]. Furthermore, during the feeding process, aphids release different kinds of saliva with different compositions and functions. This saliva may contain viruses that can be thus easily transferred to the host plant. Aphids are, therefore, organisms that can acquire and inoculate any viral taxon within plants, whatever their tissue specificity [4]. Although in general terms, it is very difficult to give a precise assessment of the potential economic loss due to aphids, many of the 275 viruses transmitted by aphids cause diseases of major economic importance [5].

Plant protection against aphid pests is dominated by synthetic insecticides, particularly those derived from active substances of the following classes: pyrethroids, neonicotinoids, carbamates, and insect growth regulators [6]. Chemical insecticides entered their golden age in the 1930s with the development of a system of synthesis and screening that allowed a biological function to be assigned to the many compounds of unknown biological activity that industry had already isolated, and this system, of course, also enabled the search for new synthetic products functioning as insecticides [7]. The extensive use of synthetic pesticides for decades uncovered a third problem arising from aphid physiology: the emergence of insecticide resistance. At least seven independent mechanisms of resistance have been described in the peach potato aphid (*Myzus persicae* (Sulzer, 1776)) [8].

In view of the above arguments, the need to look for nature-based solutions or more sustainable alternatives to chemical insecticides is much more justified. The EU has promoted the registration of low-risk substances for pest control through Directive 2009/128/EC, under which member states must ensure the rational use of plant protection products integrated with other practices and control measures, including general principles of integrated pest management (IPM) [9]. Our group has been developing solutions based on IPM for the control of aphids in Mediterranean crops cultivated in greenhouses. Thus, the predatory syrphid *Sphaerophoria rueppellii* (Wiedermann, 1820) (Diptera: Syrphidae) has been shown to be a very effective biological control agent against greenhouse aphid pests as well as an effective pollinator [10,11]. In addition to biological control, the development of control solutions based on essential oils that can enhance the effects of biological control, and thus be compatible with IPM, has become a necessity. Essential oils (EOs) are highly volatile compounds obtained from raw plant material by hydrodistillation, steam distillation, dry distillation, or mechanical cold pressing, in this latter case, for citrus fruits [12,13,14]. Chemically, EO are complex mixtures of substances classified into two main chemical groups: terpenoids, mainly monoterpenes and sesquiterpenes; and phenylpropanoids [15]. EOs are particularly abundant in conifers, such as Rutaceae, Umbelliferae, Myrtaceae, Lamiaceae, and Lauraceae [16]. And why are EOs interesting in the field of biopesticides? Many studies have shown the insecticidal effects of EOs, and an increasing number of other studies support the view that there is significant EO tolerance among specific nontarget organisms [6,17]. Also, due to their low mammalian toxicity, EO are generally recognized as safe (GRAS) by the US Food and Drug Administration [18]. In addition, the complex mixtures of substances contained in EOs showing different action mechanisms and potential synergisms may be effective in preventing the development of resistances in pest populations [15].

Thus, the interest in EO as biopesticides is not surprising, despite the difficulties in developing commercially cost-effective biopesticides based on EOs. The main difficulties have been identified elsewhere [15,19,20]. These include strict legislation, resource availability, short persistence of the compounds in the environment and a much higher than expected variability in the final EOs. Effectively, the production of a standardized product, which is important for regulatory and marketing purposes, is still challenging. In addition to the recognition of well-established chemotypes, a given chemotype growing in populations subject to specific environmental conditions may render great differences, not only in minority but also in majority components, thus substantially impacting the biological activity of these EOs [12]. Moreover, the variability of an EO composition is also impacted by the method of extraction employed [16]. However, despite these drawbacks, the search of highly effective EO is very relevant as a first step to identify those mixtures that may ensure a final cost-effective formulation and, therefore, be commercially exploited. This work is aimed at the characterisation of the essential oils of three aromatic species growing in southeastern Spain (*Thymus vulgaris* L. (thyme), *Rosmarinus officinalis* L. var. ‘prostratus’ and *Lavandula dentata* L.) and the evaluation of their insecticidal activity against aphid (*Rhopalosiphum padi* (Linnaeus, 1758)) pests.

## 2. Materials and Methods

### 2.1. Plant Material and Essential Oil Extraction

The plants used in this study were collected from Torretes Biological Station located (38°38′10.91″ N, 0°32′14.35″ W) in Ibi (Alicante, Spain) between March and April 2022. Three species belonging to the Lamiaceae family were selected: *Thymus vulgaris* L.; *Rosmarinus officinalis* L. var. ‘prostratus’ and *Lavandula dentata* L. The aerial parts of the species collected were air-dried for 6 days inside the laboratory. Then, the leaves were separated from stems and other materials and crushed in an electrical grinder. Around 50 g of ground leaves were subjected to hydrodistillation in a Clevenger-type apparatus for 3 h with distilled water at a ratio of 10 mL per gram of plant dry matter. The resulting oil fraction was recovered, dehydrated with anhydrous sodium sulphate, and stored at 4 °C in amber glass vials until utilization. The oil yield was calculated and expressed as a volume percent (*v*/*w*) calculated relative to 100 g of dry material.

### 2.2. GC-MS Characterization

The essential oils were analysed by GC-MS using an Agilent 7890A gas chromatograph coupled to an Agilent 5975C mass spectrometer. Separation was carried out in a HP-5MS capillary column (30 m length, 0.25 mm inner diameter, and 0.25 mm film thickness, Agilent). The GC oven temperature ramp initiated at 80 °C for 3 min; then the temperature was raised until 220 °C at 4 °C/min. The final temperature was maintained for 10 min. The temperature of the injector was kept at 280 °C operating in split mode (1:100 ratio). The carrier gas was helium at a rate of 1 mL/min. The mass spectrometer was equipped with an electron impact (EI) ionization mode working at 70 eV. Full-scan mass spectra were recorded in the range *m/z* 30 to 550 amu. The identification of the individual compounds was based on the comparisons of the obtained spectra with those of reference compounds available in the NIST (National Institute of Standard and Technology) v11 library by selecting a matching score above 75%. The relative abundance of components, expressed in percentage, was calculated from the peak areas of each chromatogram without calculating a response factor. The assignation of the identified compounds to lipid families was made according to Brite Hierarchy (KEGG) “https://www.genome.jp/kegg-bin/show_brite?htext=br08002%2ekeg&selected=none&extend=B61 (accessed on 22 May 2023)”. The data presented in tables are the average of the two injections ± standard deviation.

### 2.3. Insect Rearing

Bird cherry oat aphids (*Rhopalosiphum padi* L.) were supplied by Bionostrum Pest Control, S.A. (Alicante, Spain). The aphid colonies were maintained on barley (*Hordeum vulgare* L.) plants in a growth chamber at 24 ± 2 °C, 60% RH and a 14 L:10 D photoperiod provided by white LED lamps. Trays with aphid-bearing plants were covered with mesh cages (30 × 60 × 40 cm) to prevent their escape.

### 2.4. Insecticidal Activity of EO

To demonstrate the insecticidal activity of the EO, contact toxicity bioassays were performed. For this, a barley leaf was placed in a 70 mm diameter Petri dish with a pair of moistened filter paper disks at the bottom. Then, 10 apterous adults of *Rhopalosiphum padi* were placed on the leaf, left to settle for 10 min, and then the EO to be tested was sprayed over the aphids. For the bioassays, all the EO were prepared at three doses: 4, 10 and 15 μL/mL in acetone. A total volume of 1 mL was prepared for each EO and completely sprayed over the aphids with the aid of 2 mL glass spraying devices with an atomizer. Similar treatments with only acetone were used as controls. Once treatments were completed, the Petri dishes were placed into a growth chamber at 24 ± 2 °C and 60% RH. Mortality was recorded after 24 h. Contact toxicity bioassays were replicated three times. Each replicate consisted of ten Petri dishes with 10 aphids each. The data presented in tables are the average of three replicates ± standard deviation. LC_50_ was calculated by Probit analysis [21] using a Probit analysis spreadsheet calculator [22].

### 2.5. Statistical Analysis

Statistical significance was revealed by one-way ANOVA and Tukey test HSD at *p* ≤ 0.05, using R software ver. 4.2.3 [23].

## 3. Results

### 3.1. Characterization of the Essential Oils (EO)

**Figure 1:**
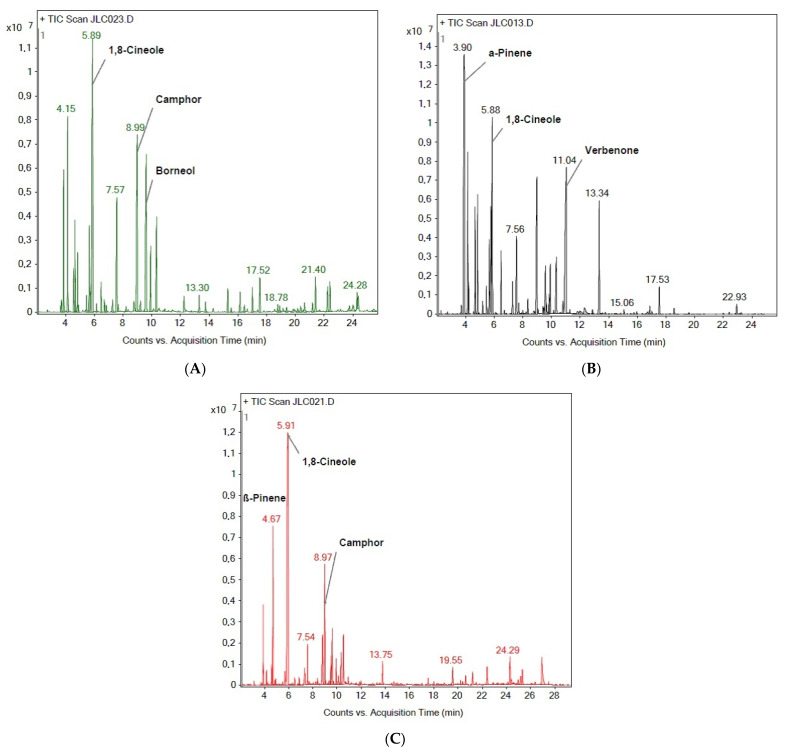
Examples of the chromatograms obtained for each of the three species studied. The three most abundant components are annotated in: (**A**) *Thymus vulgaris*; (**B**) *Rosmarinus officinalis* var. ‘prostratus’; and (**C**) *Lavandula dentata*.

The characterization of each EO studied was carried out by GC-MS. Figure 1 shows an example of the chromatograms obtained for each EO.

Essential oil extraction by hydrodistillation with the aid of a Clevenger-type apparatus for 3 h gave a yield of essential oil of 1.86% for *T*. *vulgaris*, 0.64% for *R*. *officinalis* and 2.10% for *L*. *dentata* (Table 1). The GC-MS analysis of the hydrodistilled fraction from each species revealed, in all cases, the absolute predominance of monoterpenoids, followed by sesquiterpenoids and, at far lower levels, representatives of up to 26 other chemical families, according to the species (Table 1).

**Figure 2:**
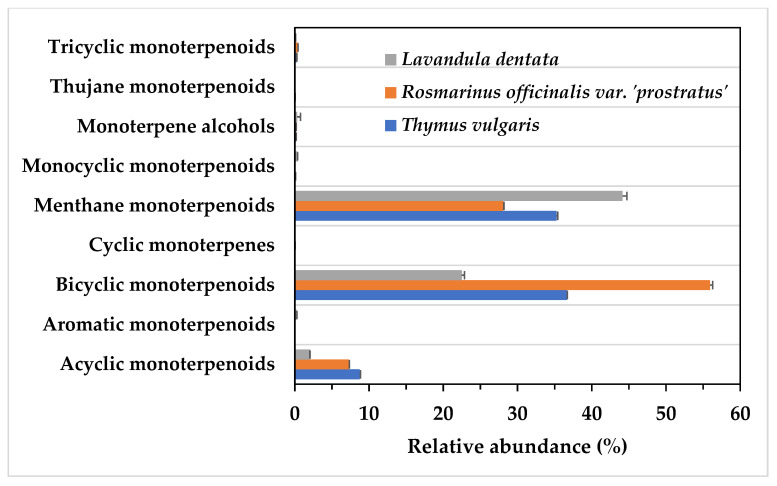
Monoterpenoid profile of the three species studied.

Monoterpenoids comprised 81.2% of the identified compounds in *T*. *vulgaris*, 91.98% in *R*. *officinalis*, and 69.74% in *L*. *dentata*, (Table 1). *T*. *vulgaris* EO contained almost equal amounts of menthane and bicyclic monoterpenoids, these two being the most abundant monoterpenoid classes found in this species (Figure 2). In *R*. *officinalis*, bicyclic monoterpenoids were, however, the major fraction, while in *L*. *dentata*, menthane monoterpenoids predominated (Figure 2).

**Figure 3:**
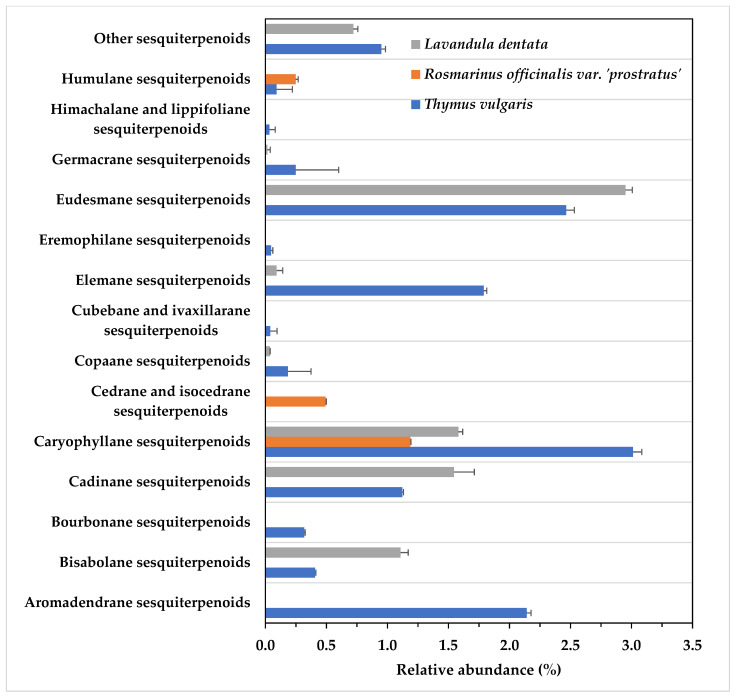
Sesquiterpenoid profile of sesquiterpenoid of the three species selected.

Sesquiterpenoids were the second most abundant fraction in the EO of the three species (Table 1), again with large variations in their respective profiles (Figure 2). Thus, the EO of *R*. *officinalis* presented a less abundant (1.93%, Table 1) and chemically diverse sesquiterpenoid fraction, with only three classes represented: caryophyllene sesquiterpenoids, cedrane and isocedrane sesquiterpenoids, and humulane sesquiterpenoids (Figure 2). The EO of *L*. *dentata* consisted of 8.05% sesquiterpenoids (Table 1) distributed into seven classes, the most abundant being eudesmane sesquiterpenoids (Figure 3). Finally, *T*. *vulgaris* EO contained the highest (12.85%, Table 1) and most chemically diverse sesquiterpenoid profile, consisting of 13 classes, with caryophyllane and eudesmane sesquiterpenoids being the most abundant classes (Figure 3).

Together, monoterpenoids and sesquiterpenoids accounted for 94% of the identified compounds in *T. vulgaris*. The rest belonged to 10 different chemical classes, and except for dimethoxybenzenes, all were above 1% (Table 1). In *R*. *officinalis*, monoterpenoids and sesquiterpenoids also accounted for almost 94% (93.91%) of the total, but the rest of the components belonged to 17 different chemical classes. In *L*. *dentata*, significantly fewer compounds (80.20%) were identified, of which 77.79% were monoterpenoids and sesquiterpenoids, and the rest also represented 17 different chemical classes, although not exactly the same as rosemary (Table 1).

A total of 84–88 compounds were identified in the EO of *T*. *vulgaris,* 61–66 in *R. officinalis*, and 72–78 in *L*. *dentata*. The ten most abundant compounds of each species are presented in Table 2, while the complete profile is available in the Supplementary Material.

### 3.2. Insecticidal Activity of EO

The insecticidal activity of the EOs was tested by contact toxicity bioassays, as described in the Materials and Methods section. The results indicate that *T*. *vulgaris* and *R. officinalis* EOs had a significant insecticidal effect on bird cherry oat aphid *Rhopalosiphum padi* in a dose-dependent manner (Table 3). *T*. *vulgaris* EO showed the highest insecticidal activity, reaching 78.3% mortality at the higher dose tested (15 μL/mL), while *R*. *officinalis* EO reached 56.7% aphid mortality at the same dose. The EOs of the two species showed an LD_50_ of 8.86 and 11.72 μL/mL, respectively. In the case of *L*. *dentata*, a non-linear response was found for all three doses tested (Table 3). The mortality was 54.7% at 10 μL/mL but decreased to 48% at the highest dose tested. The unavailability of additional amounts of this EO prevented us from calculating the LD_50_ for this species in this experiment.

Different combinations of EOs were also tested to see if blends improved the results obtained with the individual oils. For this, binary combinations of EO were prepared at 10 μL/mL each and the resulting mortality rates are presented in Table 4.

## 4. Discussion

The Eos of three Lamiaceae species, *Thymus vulgaris*, *Rosmarinus officinalis* var. ‘prostratus’, and *Lavandula dentata*, were characterized and tested for their potential utilization as biopesticides against aphids. *T*. *vulgaris* L. is probably the most commercially used species among the 215 accepted species of genus *Thymus* [24]. Three subspecies, *vulgaris*, *aestivus* and *palearensis*, are recognized within this genus, mainly based on the chromosome number, but the frequent presence of hybrids adds further complexity to this group [24]. In the present work, the menthane monoterpenoid 1,8-cineole (17.8%) was reported as the major compound present in *T*. *vulgaris* EO, followed by two bicyclic monoterpenoids, camphor (11.1%) and borneol (10.3%), heading a list of more than 80 identified compounds (see Supplemental Material). This chemotype with 1,8-cineole as the majority component is considered endemic to the Iberian Peninsula [25]. In a study monitoring variations in the EO composition with phenological stages of Spanish thyme, the authors found 1,8-cineole (36.42%) followed by terpenyl acetate (18.17%) and borneol (4.77%) to be the major components, with the former two changing significantly during the whole vegetative cycle of the plant [25]. Chemical variation in essential oil composition also comes from cultural practices. Thus, volatile components of *Thymus vulgaris* L. from wild-growing and cultivated plants in Jordan were found to vary not only in oil yield but also in the relative abundance of major EO components according to the population location in the case of plants growing wild, but also with respect to cultivated plants. Carvacrol (85.2–86.1%) was the most abundant component in two wild populations, while thymol was the majority (63.8%) in the third wild population studied, clearly corresponding to carvacrol and thymol-type chemotypes. Interestingly, in the cultivated thyme, thymol and carvacrol were present in substantial quantities (30.7% and 50.6%, respectively) [26]. Undoubtedly, this extreme variation in individual components of essential oils according to phenological stage, cultural practice, or environmental conditions, hinders the roadmap towards the industrial exploitation of essential oils.

As shown in Table 3, *T*. *vulgaris* EO showed increasing aphid mortality with dose in contact toxicity bioassays, reaching a maximum toxicity of 78.3% and LD_50_ of 6.47 μL/mL (6.47 mL/L). This response allows us to consider this thyme EO as promising for its further development as a botanical insecticide against aphids and supports the widely alleged insecticidal properties of thyme EO. For instance, 3 mL of *T*. *vulgaris* EO provoked 85% mortality after 24 h in toxicity bioassays against two stored grain pest species, the rice weevil *Sitophylus oryzae* (Linnaeus, 1763) (Coleoptera: Curculionidae) and the lesser grain borer *Rhysopertha dominica* (Fabricius, 1792) (Coleoptera: Bostrichidae,), and this mortality rate increased up to 100% after 48 h of treatment [27]. In this case, the *T*. *vulgaris* EO was characterized by the following major components: carvacrol (27.31%), thymol (21.51%), and γ-terpinene (19.17%) [27]. A *T*. *vulgaris* EO with thymol (47.19%), *p*-cymene (28.37%) and γ-terpinene (8.06%) as major components showed fumigant activity against the green stink bug *Nezara viridula* Linnaeus, 1758 (Hemiptera: Pentatomidae) with an LC_50_ of 8.9 mg/mL for nymphs and 219.2 mg/mL for adults [28]. With very similar results regarding the major components of their *T*. *vulgaris* EO, LC_50_ values of 57.48 and 75.80 mg/L after 72 h of treatment were reported against the planthopper *Pochazia shantungensis* (Hemiptera, Ricaniidae) nymphs and adults, respectively [29]. EOs from *T. vulgaris* have been successfully used as preservatives against postharvest fungal infestation of food commodities [30], although the authors gave no characterization of the components of the extracted EO. Focusing on the specific fight against aphids, we have not found previous reports on the effects of *T*. *vulgaris* EO on aphids. However, a maximum mortality of 99.1% of *Foeniculum vulgare* Mill. seeds EO was reported against *Myzus persicae* [31]. Two EO preparations from two *Mentha pulegium* L. chemotypes gave 86.2% and 88% mortality, respectively, against the cotton aphid *Aphis gossypii* Glover, 1877, but 40–48% against *Aphis spiraecola* Patch,1914, although in this last case, the dosage applied was half that used with *A*. *gossypii* [32]. In line with the results obtained in the present work, LC_50_ values of 6.62 mL/L and 2.04 mL/L in contact toxicity bioassays with *Aphis gossypii* were reported using EO from yarrow (*Achillea wilhelmsii* K.Koch) and sweet asafetida (*Ferula assa*-*foetida* L.), respectively [33].

Rosemary (*Rosmarinus officinalis* L.) EO has also been frequently used in traditional medicine, mainly in the Mediterranean basin, where it is native. The reported biological effects of rosemary EO include antioxidative, anti-inflammatory, anticarcinogenic, spasmolytic, antibacterial, and antifungal activities, as well as many others [34,35]. As in the case of thyme, rosemary EO shows high intraspecific chemical variability according to geographical origin, environmental and/or agronomic conditions, harvest time or extraction method [36], and different chemotypes defined according to the major component of rosemary EO have also been proposed [37]. In this work, the variety ‘prostratus’ of *Rosmarinus officinalis*, long recognised in gardening but much less known from a phytochemical and biological activity point of view, was tested. This cultivar is appreciated for its fast establishment and soil coverage, features which keep weeds under control and thus reduce the use of chemicals in Mediterranean urban landscapes [38]. *R*. *officinalis* var. ‘prostratus’ EO was characterized by the bicyclic monoterpenoids α-pinene (18.74%) and verbenone (13.42%) and by the menthane monoterpenoid 1,8-cineole (10.32%) (Table 2). Ref. [39] studied the EO composition and yield of a natural population of *R. officinalis* in Sardinia (Italy) with an extended flowering period (from September to May). They also identified α-pinene as the major component, although at a much higher percentage (48.03–75.4%), followed by 1,8-cineole (7.30–15.65%), caryophyllene (1.80–12.59%), borneol (1.00–5.16%), camphene (2.02–4.27%) and verbenone (1.04–5.84%), the intervals representing the range of each compound through the entire year. In addition to factors such as genetic background or environmental conditions, the EO extraction process itself influences the composition of the EO. Thus, comparing the extraction of rosemary oil by steam distillation and hydrodistillation revealed important differences: Hydrodistillation gave a component profile in which 1,8-cineole (31.9%), camphor (19.7%), α-terpineol (12.8%), and borneol (12.1%) were the most abundant components, while 1,8-cineole (52.4%), camphor (12.6%), β-pinene (5.7%) and α-pinene (5.2%) were the majority compounds with steam distillation [40].

The mortality rate achieved using *R*. *officinalis* var. ‘prostratus’ EO reached 56.7% with 15 μL/mL (LD_50_ 11.72 μL/mL) (Table 3). This is a significantly lower mortality rate than that obtained with thyme EO, but also suggests it as a promising potential biopesticide. Similar mortality rates were reported by [41], whose analysis of *R*. *officinalis* EO, among other plant species, revealed 1,8-cineole (35.27%), α-pinene (15.87%), and camphor (10.43%) as the most abundant components. They found 60% mortality at 24 h against *Myzus persicae* in contact toxicity assays. EO from *R*. *officinalis* has also been tested against other pests. For instance, LC_50_ was achieved with 10.0 mL/L of rosemary EO against adult females of the red spider mite *Tetranychus urticae* C.L. Koch, 1836 reared on bean plants, and with 13.0 mL/L for those reared on tomato plants [42]. The major components of this rosemary oil were 1,8-cineole (31.5%), camphor (20.0%) and α-pinene (17.5%), and complete mortality of mites was obtained with 20 mL/L of the oil on bean plants and 40 mL/L on tomato plants [42]. Mortalities of 95–100% of *Tetranychus urticae* were reported by a rosemary EO characterized by 1,8-cineole (26.7%), α-pinene (18.5%) and camphor (17.5%) [43]. Another *R*. *officinalis* EO characterized by camphor (31.16%), β-caryophyllene (18.55%) and 3,4-dimethyl-2,4-hexadiene (9.08%) as the most abundant components showed biological activity against the flour beetle *Tribolium confusum* Jacquelin du Val, 1863 (Coleoptera: Tenebrionidae), finding complete mortality with 0.12 μL/mL [44].

*Lavandula* is a genus comprising 39 species and around 400 registered cultivars [45]. *Lavandula dentata* L., the species used in the present work, has also been widely employed in traditional medicine and has shown biological activity, including antifungal, antioxidant, antibacterial and antiparasitic activities [46]. In our case, *L*. *dentata* EO was characterized by 1,8-cineole (34.65%), camphor (7.58%) and β-pinene (6.39%) as major components giving a maximum mortality of 54.7% at 10 μL/mL on *Rhopalosiphum padi*. It was reported by [47] that the EO of *L*. *dentata* was composed mainly of 1,8-cineole (34.33%), fenchone (17.78%) and camphor (15.75%). This EO showed good insecticidal activity in assays carried out without insect food against three pest insects: the maize weevil *Sitophilus zeamais* (Motschulsky, 1855) (Coleoptera: Curculionidae) (LC_50_ 26.9 μL/L air), the red flour beetle *Tribolium castaneum* (Herbst, 1797) (Coleoptera: Tenebrionidae) (LC_50_ 11.3 μL/L air), and *Epicauta atomaria* (Germar, 1821) (Coleoptera: Meloidae) (LC_50_ 26.9 μL/L air). For *S*. *zeamais* and *T*. *castaneum*, the authors found significantly higher LC_50_ (42.7, and 29.3 μL/L air, respectively) when the assays were conducted with food. *L*. *dentata* flowering tops collected from northern Tunisia were characterized by β-eudesmol (21.18%), myrtenol (13.02%), and sabinol (11.02%) as major compounds. In addition to antifungal and antibacterial activities, this EO showed strong fumigant, repellent, and contact properties against *Rhyzopertha dominica* Fabricius, 1792 (Coleoptera: Bostrichidae) and *Sitophilus oryzae* (Linnaeus, 1764) (Coleoptera: Curculionidae) [48].

Collectively considered, the results presented in this paper show promising activity against aphids (*Rhopalosiphum padii*) of the EO from three Lamiaceae species, particularly *T*. *vulgaris*, evaluated in terms of mortality in toxicity bioassays. The EOs from the three species caused over 50% mortality in aphids at any of the doses used, and with *T*. *vulgaris* EO, this mortality was close to 80%. Interestingly, in two of the species studied, *T*. *vulgaris* and *L*. *dentata*, 1,8-cineole was found to be the major compound in their respective EO, but nevertheless, the mortality results were very different. This supports the view that the bioactivity of an EO is not strictly related to its majority components, but to some kind of synergy between the majority and minority components [49]. This is also evident from the results with the EO combinations (Table 4). Although only one dose (10 mL/L) of binary EO combinations was tested, those mixtures that included *T*. *vulgaris* EO performed worse than this EO alone. Conversely, *L*. *dentata* EO increased mortality when applied in mixtures, compared with the same dose of the single oil.

In conclusion, the EO composition of three Lamiaceae species were characterized as a first step in their possible utilization in biopesticide formulations. Comparing the three species and according to the data obtained, *Thymus vulgaris* EO proved to be the most effective against aphids.

## Figures and Tables

**Table 1:** Characterization of the essential oils of the three species selected.

Compound Family	*T. vulgaris*	*R. officinalis* var. ‘Prostratus’	*L. dentata*
Monoterpenoids	81.20 ± 0.23	91.98 ± 0.23	69.74 ± 0.50
Sesquiterpenoids	12.85 ± 0.21	1.93 ± 0.03	8.05 ± 0.33
Acetophenones			0.06 ± 0.002
Alcohols			0.02 ± 0.03
Aldehydes		0.23 ± 0.03	0.08 ± 0.09
Anisols	0.05 ± 0.00003	0.01 ± 0.0001	
Aromatic hydrocarbons		0.01 ± 0.0005	0.03 ± 0.04
Benzoic acid esters		0.06 ± 0.04	
Benzoyl derivatives		0.01 ± 0.0004	0.03 ± 0.002
Branched unsaturated hydrocarbons	0.13 ± 0.18	0.04 ± 0.03	0.02 ± 0.02
Cetones			0.34 ± 0.02
Cycloalkenes		0.02 ± 0.03	
Cyclodienes			0.12 ± 0.17
Dimethoxybenzenes	1.00 ± 0.01	0.11 ± 0.001	0.10 ± 0.02
Diterpenoids		0.07 ± 0.02	0.40 ± 0.01
Enones		0.06 ± 0.002	
Esters	0.23 ± 0.001	0.01 ± 0.02	0.35 ± 0.03
Fatty alcohols	0.03 ± 0.0004		0.02 ± 0.0002
Fatty esters	0.09 ± 0.001	0.02 ± 0.01	0.40 ± 0.01
Jasmonate esters		0.02 ± 0.0004	
Medium-chain aldehydes	0.07 ± 0.0004	0.05 ± 0.001	0.01 ± 0.01
Naphthalene			0.05 ± 0.07
Octadecanoids		0.32 ± 0.002	
Phenols			0.13 ± 0.001
Phenylacetaldehydes		0.06 ± 0.0001	0.20 ± 0.003
Phenylpropanoids	0.18 ± 0.003	0.02 ± 0.001	
Polycyclic hydrocarbons	0.04 ± 0.06		
Tertiary alcohols	0.14 ± 0.20		
Unclassified	3.97 ± 0.16	4.97 ± 0.36	19.8 ± 0.22
Total identified (%)	96.03 ± 0.16	95.03 ± 0.36	80.20 ± 0.23
Yield (mL EO 100 g dw^−1^)	1.86 ± 0.51	0.65 ± 0.17	2.10 ± 0.75

**Table 2:** Ten most abundant compounds of the essential oils of the three species analysed.

Compound	R.I. ^a^	Relative Abundance (%)
*Thymus vulgaris*		
1,8-Cineole	1035	18.11 ± 0.46
Camphor	1148	11.18 ± 0.05
endo-Borneol	1168	10.32 ± 0.02
Camphene	953	5.81 ± 0.04
Linalool	1102	5.46 ± 0.04
α-Terpineol	1194	4.54 ± 0.03
α-Pinene	940	3.74 ± 0.04
*o*-Cymene	1027	3.55 ± 0.04
4-Terpineol	1179	2.73 ± 0.01
β-Pinene	982	2.66 ± 0.03
*Rosmarinus officinalis* var. ‘prostratus’		
α-Pinene	940	18.72 ± 0.15
Verbenone	1216	13.42 ± 0.03
1,8-Cineole	1035	10.32 ± 0.002
Camphor	1148	7.14 ± 0.03
Bornyl acetate	1286	5.17 ± 0.01
Limonene	1031	4.83 ± 0.05
Camphene	953	4.41 ± 0.04
β-Myrcene	992	3.47 ± 0.05
Linalool	1102	3.02 ± 0.01
α-Terpineol	1194	3.01 ± 0.02
*Lavandula dentata*		
1,8-Cineole	1035	34.65 ± 0.95
Camphor	1148	7.58 ± 0.07
β-Pinene	982	6.39 ± 0.09
Myrtenal	1197	3.16 ± 0.01
α-Pinene	940	2.56 ± 0.03
α-Terpineol	1194	1.91 ± 0.01
Linalool	1102	1.86 ± 0.002
β-Eudesmol	1651	1.76 ± 0.03
*p*-Thymol	1303	1.50 ± 0.02
Pinocarvone	1164	1.43 ± 0.001

^a^ Linear retention index (R.I.) experimentally calculated using standard C_7_–C_40_ alkanes.

**Table 3:** Mortality of *Rhopalosiphum padi* in contact toxicity bioassays with the selected EO.

Essential Oil	Dose (μL/mL)	Mortality (%) Mean ± SD *	LD_50_ (μL/mL) (95% CI) ^+^
*Thymus vulgaris*	4	34.3 ± 23.3 ^a^	6.47 (4.01–10.42)
	10	54.3 ± 22.9 ^a^	
	15	78.0 ± 24.6 ^b^	
*Rosmarinus officinalis* var. ‘prostratus’	4	38.6 ± 21.7 ^a^	9.94 (2.85–34.60)
	10	41.7 ± 25.3 ^a^	
	15	56.7 ± 25.6 ^b^	
*Lavandula dentata*	4	46.9 ± 25.4 ^a^	-
	10	53.7 ± 24.8 ^b^	
	15	31.2 ± 21.6 ^c^	
Control		14.2 ± 11.4	

* Means with different letters indicate significant differences for each essential oil (*p* ≤ 0.05); ^+^ Lethal dose (LD_50_) (Confidence Interval (CI)).

**Table 4:** Mortality of *Rhopalosiphum padi* in contact toxicity bioassays with mixtures of EOs.

Essential Oil Binary Mixture ^1^	Mortality (%) Mean ± SD *
*T. vulgaris* + *R*. *officinalis* var. ‘prostratus’	41.2 ± 21.4 ^a^
*R*. *officinalis* var. ‘prostratus’ + *L*. *dentata*	62.7 ± 28.5 ^b^
*T*. *vulgaris* + *L*. *dentata*	61.5 ± 26.3 ^b^

^1^ All EO were used at 10 μL/mL. * Means with different letters indicate significant differences between mixtures (*p* ≤ 0.05).

## Data Availability

All data supporting the present article is contained in supplementary material.

## Supplementary Materials

The following supporting information can be downloaded at: https://www.mdpi.com/article/10.3390/life13081621/s1.
